# Effectiveness of feedback with a smartwatch for high-quality chest compressions during adult cardiac arrest: A randomized controlled simulation study

**DOI:** 10.1371/journal.pone.0169046

**Published:** 2017-04-03

**Authors:** Chiwon Ahn, Juncheol Lee, Jaehoon Oh, Yeongtak Song, Youngjoon Chee, Tae Ho Lim, Hyunggoo Kang, Hyungoo Shin

**Affiliations:** 1 Department of Emergency Medicine, College of Medicine, Hanyang University, Seoul, Korea; 2 Department of Biomedical Engineering, Graduate School of Medicine, Hanyang University, Seoul, Korea; 3 Convergence Technology Center for Disaster Preparedness, Hanyang University, Seoul, Korea; 4 School of Electrical Engineering, University of Ulsan, Ulsan, Korea; Azienda Ospedaliero Universitaria Careggi, ITALY

## Abstract

Previous studies have demonstrated the potential for using smartwatches with a built-in accelerometer as feedback devices for high-quality chest compression during cardiopulmonary resuscitation. However, to the best of our knowledge, no previous study has reported the effects of this feedback on chest compressions in action. A randomized, parallel controlled study of 40 senior medical students was conducted to examine the effect of chest compression feedback via a smartwatch during cardiopulmonary resuscitation of manikins. A feedback application was developed for the smartwatch, in which visual feedback was provided for chest compression depth and rate. Vibrations from smartwatch were used to indicate the chest compression rate. The participants were randomly allocated to the intervention and control groups, and they performed chest compressions on manikins for 2 min continuously with or without feedback, respectively. The proportion of accurate chest compression depth (≥5 cm and ≤6 cm) was assessed as the primary outcome, and the chest compression depth, chest compression rate, and the proportion of complete chest decompression (≤1 cm of residual leaning) were recorded as secondary outcomes. The proportion of accurate chest compression depth in the intervention group was significantly higher than that in the control group (64.6±7.8% versus 43.1±28.3%; *p* = 0.02). The mean compression depth and rate and the proportion of complete chest decompressions did not differ significantly between the two groups (all *p*>0.05). Cardiopulmonary resuscitation-related feedback via a smartwatch could provide assistance with respect to the ideal range of chest compression depth, and this can easily be applied to patients with out-of-hospital arrest by rescuers who wear smartwatches.

## Introduction

The new 2015 American Heart Association (AHA) guidelines recommend rescuers to perform compressions to a depth of 5–6 cm and at a rate of 100–120 counts/min; this is in contrast to the previous guidelines, which recommended a depth of at least 5 cm and a rate of 100 counts/min [1, 2]. This range of chest compression (CC) depth is sufficient to achieve an effective outcome without the development of complications, including fracture of the ribs, pneumothorax, and hemothorax, which are associated with a compression depth of >6 cm [3–5]. In addition, compressions at a rate of >120 compressions/min might have result in poor coronary perfusion and low cardiac output by reducing the diastolic filling time [6]. It is often challenging for rescuers to reach the recommended range of CC depth during cardiopulmonary resuscitation (CPR). CPR skills can be improved by regular training [7]; however, rescuers cannot be expected to retain CPR skills after a certain period of time owing to a decline in learning effects [8–10]. A CPR feedback system that provides accurate information in real-time could overcome this problem in real-life situations involving cardiac arrest.

In previous studies, various feedback devices with a built-in accelerometer and pressure sensor could provide data regarding the CC depth, rate, and intensity [11–19]. Rescuers who performed CC with auditory-visual feedback devices were found to have improved individual CPR performances [11–13, 17, 19]. Recently, feedback systems involving a smartphone with a built-in accelerometer have been developed [20, 21]. Such devices provide auditory-visual information to the rescuer via a speaker and screen on the device. Smartwatches as the newest wearable smart device also have a built-in accelerometer and could be useful as a CPR feedback device. In our previous study, we verified and reported an algorithm of compression depth estimation using a smartwatch, and developed a smartwatch application that was able to provide visual information on CC depth and rate in real-time [22].

Although a previous study reported on the potential use of smartwatches with built-in accelerometers as feedback devices for high-quality CC [22], no study has reported the effect of this feedback on CC during CPR. We hypothesized that the CPR feedback system of a smartwatch might improve the parameters of CC performed by rescuers who have received CPR training.

## Materials and methods

### Design

We designed a prospective, randomized controlled parallel study to evaluate the ability of a feedback application on a smartwatch to improve the quality of CC administered during simulated cardiac arrest. The study was performed at Hanyang University’s Simulation Center (Seoul, Republic of Korea) in April 2016. The study was approved by the Institutional Review Board of the Hanyang University Hospital (HYUH201512024001-HE002), and the study protocol was registered in the Clinical Research Information Service (cris.nih.go.kr: KCT0001799).

### Participants

Forty medical students from Hanyang University participated voluntarily in this study. The inclusion criteria were healthy individuals aged >18 years. Volunteers were excluded if they had wrist, spine, or pulmonary/heart diseases. Two certified Basic Life Support instructors taught all participants the high-quality curriculum of Basic Life Support, for a total of 4 hours in 2 weeks. The participants received information related to this study before the experiments. All participants provided their written informed consent.

**Fig 1:**
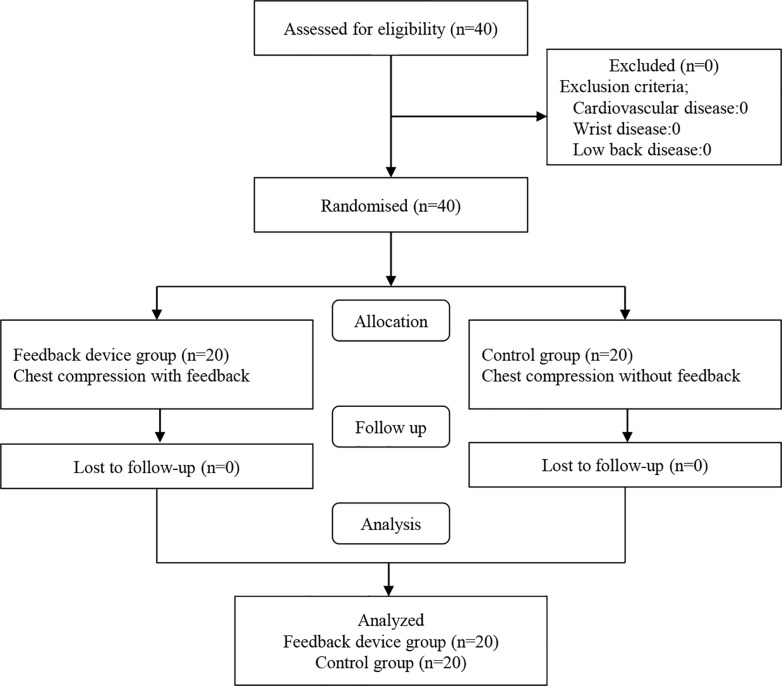
A flowchart showing participant enrolment.

A pilot study was conducted to detect a difference in the proportion of CC depth (≥5 cm and ≤6 cm) between the smartwatch feedback group and the control group. The proportion (mean±SD) of accurate CC depth was 96.8±3.2% and 69.8±30.2%, respectively. G-power 3.1.2® (Heine Heinrich University, Düsseldorf, German) was used to calculate a required sample size of 18 participants per group with an effect size of 1.25, α-error of 0.05, and power of 0.95. We aimed to recruit 40 participants to account for a possible drop-out rate of 10% (Fig 1).

### Equipment and materials

The present study used a CPR training manikin (SkillReporter™; Laerdal, Stavanger, Norway) and the manikin’s recording programme via a laptop for simulation. The manikin could estimate several parameters, including CC depth, rate, and relaxation depth via a sensor when participants compressed the middle part of the manikin’s chest. As a feedback device, a smartwatch (Galaxy Gear Live; Samsung Electronics, Seoul, Republic of Korea) was used to implement the application [22]. The experiment was performed on a flat and firm surface to avoid the mattress effect, which absorbs some of the force of the CC.

**Fig 2:**
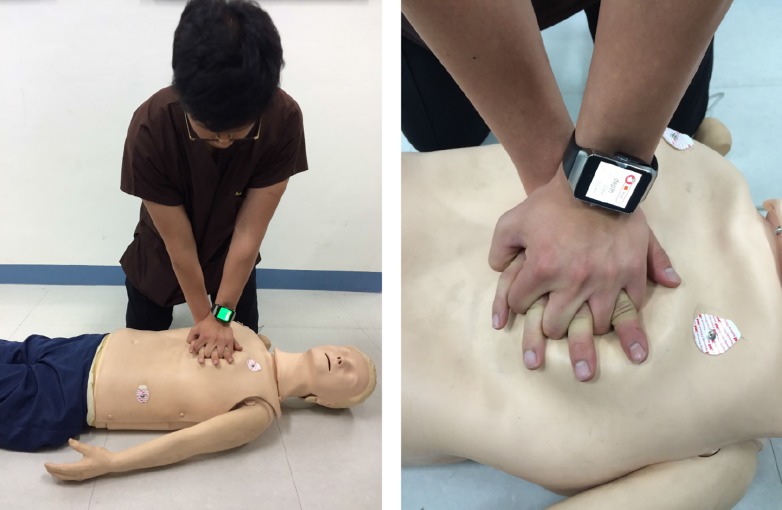
Chest compressions using a smartwatch-based feedback system. Participants, in a kneeling position adjacent to a manikin, compressed the chest of the manikin placed on a flat and firm surface. The smartwatch provided visual feedback and vibratory guidance regarding the chest compression depth and rate, respectively.

**Fig 3:**
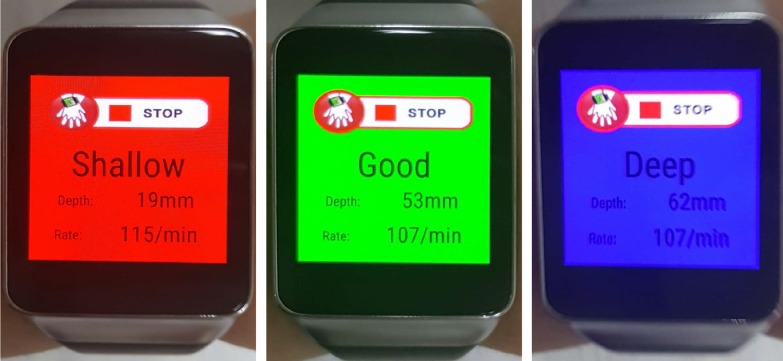
Visual feedback was given in the form of different colors according to the chest compression depth. (A) The color shown on the display of the smartwatch was red when the depth was <5 cm, (B) green, when the depth was between 5 and 6 cm, and (C) blue, when the depth was >6 cm.

The participants performed CC on a manikin while wearing a smartwatch, during which the display of the device showed three different colors as visual feedback (Fig 2). A blue color was shown on the display when the CC depth was >6 cm, whereas a red color was shown when the CC depth was <5 cm. A green color was shown when the range of the CC depth was between 5 and 6 cm (Fig 3). In addition, regular vibrations generated by the smartwatch at a rate of 110/min were used to guide the CC rate.

### Intervention

All participants were allocated in a 1:1 ratio randomly into two groups: intervention (n = 20) and control (n = 20) groups. Randomization was performed using a sequence generator (http://www.random.org/). All participants in the two groups performed CC continuously without artificial breathing in the kneeling position beside the manikin for 2 min (Fig 2). In the intervention group, participants initiated CC wearing the smartwatch, which was running the feedback program. Conversely, the participants of the control group performed CC without the aid of a feedback device. A partition was placed on the floor between investigators and a participant so that the investigators could not recognize the participants’ group during the experiment. Participant characteristics, such as age, weight, CPR education frequency, and experience of a real-life CPR situation, were evaluated. Data were downloaded and collected directly from the manikin’s recording system by one author who was blinded to the participants’ group allocation.

### Primary and secondary outcomes

In this study, the primary outcome was the proportion of accurate CC depth that was defined as the ratio of the number of CC whose depth was between 5 and 6 cm to the total number of CC in 2 min. In addition, CC depth and rate, and the proportion of complete chest decompression were investigated as secondary outcomes. The proportion of complete chest decompression was defined as the ratio of the number of decompressions whose recoil depth was <1 cm to the total number of decompressions in 2 min.

### Statistical analyses

The data were compiled using a standard spreadsheet application (Excel 2016; Microsoft, Redmond, WA, USA) and were analyzed using the Statistical Package for the Social Sciences (SPSS), version 21.0 KO for Windows (SPSS Inc., Chicago, IL, USA). The Kolmogorov-Smirnov tests were performed for normality of all dataset. Descriptive statistics were used to describe the baseline characteristics of the study participants and to present categorical variables as frequencies and percentages. Normally distributed data are presented as the mean±SD with 95% confidence intervals (CI), whereas non-normally distributed data are presented as medians with interquartile ranges (IQR) with 95% CI. Student’s t-tests and Mann-Whitney U-tests were used for comparisons of continuous variables, and Chi-square or Fisher’s exact test, for categorical variables. Analysis of covariance (ANCOVA) was performed to adjust for influencing factors [23]. A *p*-value of <0.05 was considered statistically significant.

## Results

A total of 40 participants were recruited in this study; there were no exclusions (Fig 1). The baseline characteristics of the participants are summarized in Table 1. The proportion of accurate CC depth (mean±SD) for the intervention and control groups was 64.6±7.8% and 43.1±28.3%, respectively (*p* = 0.02). There were no significant differences in the CC depth, CC rate, and proportion of complete chest decompression (Table 2).

**Table 1:** Characteristics of the intervention and control participants.

	Intervention group (n = 20)	Control group (n = 20)	*P*-value^a^
Age; years	19 [18–24]	19 [18–19]	0.398
Male	19 (95%)	15 (75%)	0.182
Height; cm	174.1±6.8	171.3±5.5	0.168
Weight; kg	69.2±9.6	64.1±9.0	0.090
Body mass index	22.9±3.2	21.8±2.6	0.250
Number of CPR training	2 [1–3]	2 [1–4]	0.221
Performance CPR in real world	0 [0–0]	0 [0–0]	-

Values are mean (SD), median [IQR] or number (proportion).

^a^*p*-value <0.05 is significant.

**Table 2:** Quality of CC performed by the intervention and control group participants.

	Intervention group (n = 20)	Control group (n = 20)	*P*-value^a^	*P*-value^a^ ANCOVA
Proportion of accurate CC depth; %	64.6±7.8	43.1±28.3	0.020	0.049
CC rate; counts/min	115.5±8.2	115.2±12.1	0.915	0.555
CC depth; mm	53.1±4.1	51.1±7.7	0.310	0.927
Proportion of complete chest decompression; %	100.0 (99.3–100.0)	100.0 (99.5–100.0)	0.366	N/A

Values are mean (SD), median [IQR], or number (proportion), and tested by the independent t-test or Mann-Whitney test. ANCOVA included age, sex, and body mass index as covariates. Proportion of accurate CC depth was defined as the ratio of the number of CC whose depth was between 5 and 6 cm to the total compression number for 2 min. Proportion of complete chest decompression was defined as the ratio of the number of CC whose recoil depth was <1 cm to the total decompression number for 2 min.

CC, chest compression; ANCOVA, analysis of covariance, N/A, not applicable

^a^*p*-value <0.05 is significant.

We performed ANCOVA to adjust for factors such as sex, age, and, body mass index in order to investigate the main factors influencing the outcomes (Table 2). The only independent factor affecting the proportion of accurate CC depth was feedback (*p* = 0.05). There were no significant differences in CC depth and rate between the intervention and control groups.

Additionally, CC depth was affected by sex (*p* = 0.02), regardless of the intervention. There was no significant influencing factor for the CC rate.

## Discussion

High-quality CPR, including the maintenance of accurate depth and rate, is major requisite for improving the survival rates of cardiac arrest patients [1, 19, 24–26]. Various studies on CPR feedback devices have shown that feedback improves the CC depth and/or rate in both novice and/or trained participants [11–13, 16–18, 27]. In this simulation study with trained participants, we have shown that the use of a feedback system using a smartwatch does not improve the mean CC depth and decompression, but it improves the proportion of accurate CC depth. Thus, a smartwatch may be a good device for providing feedback while performing CC and ensuring an accurate range of depth without any complications during CPR.

There was no significant difference in the mean CC rate between the two groups. The participants received feedback regarding the CC rate by the vibration from the smartwatch instead of an auditory signal. It might have been difficult for them to perceive the vibration because of the CC motion. Hence, it is unlikely that feedback regarding the CC rate in the form of vibrations would be as effective as auditory feedback. In further studies, we will investigate whether vibration intensity affects the ability to recognize a vibration.

Several studies have reported that female rescuers with a low body weight might find CC challenging and that physical differences between male and female rescuers might affect the CC depth [28–31]. In this study, there was a significant difference between the sexes in the mean CC depth while the existence of feedback regarding the proportion of accurate CC depth, after adjusting for influencing factors. We believe that the ability to compress the chest deeper depends on physical strength. And, feedback could be important to achieve the proper CC depth without developing complications according to the guidelines.

Recently, the use of smart devices has spread exponentially, and a feedback system based on the use of smart devices to improve the quality of CC has emerged. The effectiveness of a feedback system based on a smartphone has previously been reported in manikin studies [20, 21]. This system involves the rescuer grasping the smartphone in the palm of one hand or placing the smartphone in a pouch or pocket when performing CC. However, this could lead to errors in the acceleration measurement depending on the mounting method [21, 32]. Smartwatches might overcome the limitations of smartphones because they can be worn and they are lightweight devices that are strapped to the wrist. Thus, smartwatches could be useful feedback devices for individuals who use a smartwatch for performing CPR in real-life situations.

This study has several limitations. First, this was a simulation study using a manikin; thus, the various features in clinical environment were not considered. To address this, clinical studies on the effects of CC-related feedback are required. Second, this feedback system with an accelerometer could not compensate for the mattress compression and may be inappropriate for in-hospital cardiac arrest patients placed on a bed. Third, participants consisted of young attendees of a medical school. A previous study reported that CPR is often performed by elderly rescuers [33], which may be because the majority of cardiac arrests occur in the elderly, and it is often the responsibility of their cohabitee to perform CPR. Therefore, the age of our study population is a limitation of this study. Fourth, a cardiac arrest in real life is often urgent and a rescuer is typically in a panicked state; hence, it is important that feedback devices are operated and applied quickly. However, this study did not assess the time it took participants to operate the smartwatch feedback program.

## Conclusions

In conclusion, a smartwatch CPR feedback system could assist rescuers by providing feedback regarding the ideal range for CC depth in concordance with CPR guidelines from the AHA. A smartwatch is a good and wearable feedback device for CC during CPR, and this system could easily be applied to CPR performed for out-of-hospital cardiac arrest patients by rescuers who wear smartwatches.

## Supporting information

S1 Data (XLSX)
